# Premature Loss of Deciduous Teeth as a Symptom of Systemic Disease: A Narrative Literature Review

**DOI:** 10.3390/ijerph19063386

**Published:** 2022-03-13

**Authors:** Karolina Spodzieja, Dorota Olczak-Kowalczyk

**Affiliations:** Department of Paediatric Dentistry, Medical University of Warsaw, Binieckiego 6 Street, 02-097 Warsaw, Poland; dorota.olczak-kowalczyk@wum.edu.pl

**Keywords:** tooth loss, toothlessness, oral health, general health, systemic disease, pediatric dentistry, special needs dentistry, deciduous dentition, disorders of tooth loss

## Abstract

Background: Premature loss of primary teeth can occur as a consequence of dental trauma, neonatal tooth extraction, early childhood caries, or periodontal problems, or it can be a manifestation of systemic disease. This review aims to present systemic disorders that can lead to premature loss of deciduous teeth in children and to provide a comprehensive resource for clinical practice for both physicians and dentists. Methods: This study is a narrative review of original studies and case reports published in English and Polish between 1957 and 2021 that was conducted by searching electronic scientific resources: PubMed, Google Scholar, Web of Science, and Science Direct. The schema of the qualification process is represented by a Preferred Reporting Items for Systematic Reviews and Meta-Analyses (PRISMA). In total, 196 articles were identified; after provisional assessment of the titles and abstracts by two reviewers, 46 were found to be relevant to the topic, including 1 review, 16 original papers, and 27 case reports regarding systemic disease resulting in premature tooth loss. Results: In this study, 16 systemic diseases were linked to premature primary tooth loss in children: Papillon–Lefèvre syndrome, mucocutaneous dyskeratosis, Coffin–Lowry syndrome, congenital adrenal hyperplasia, Langerhans cell histiocytosis, cherubism, hypophosphatasia, acatalasia, Chediak–Higashi syndrome, cyclic neutropenia, erythromelalgia, Down syndrome, Hajdu–Cheney syndrome, short bowel syndrome, leukocyte adhesion deficiency type 1 (LAD-1), and Wiedemann–Steiner syndrome (WSS).

## 1. Introduction

Deciduous dentition is responsible for chewing, speech and language abilities, and stimulating the growth of the stomatognathic system. Early tooth loss or premature exfoliation of teeth is defined as the loss of teeth in the oral cavity before the normal expected period. Both local and systemic factors can contribute to this phenomenon [1]. Premature tooth loss can occur as a consequence of dental trauma, neonatal tooth extraction, early childhood caries, or periodontal problems, or it can be a manifestation of systemic disease. The most common causes of premature tooth loss are dental caries and trauma, but when these are excluded, it may be difficult to reach a diagnosis due to the lack of experience of dental practitioners. Premature loss of primary teeth can cause orthodontic problems such as crowding, ectopic eruption, or tooth impaction, which can result in malocclusion. It can also affect children’s phonation, causing speech distortion. Psychosocial problems can also arise from premature tooth loss in children, especially when the child loses anterior teeth. Although younger children may not understand how missing a tooth affects their quality of life, some of them may feel unattractive in comparison to other children. However, there is a lack of scientific data concerning the consequences of premature primary tooth loss in children; therefore, this topic needs to be further investigated.

Unfortunately, because many dental practitioners are unaware of the systemic conditions that can lead to premature primary tooth loss, and because of incorrect diagnosis or no diagnosis at all, the proper treatment cannot be implemented. Since there is a significant lack of scientific reviews concerning this topic, the aim of this study was to gather and present systemic diseases that may cause premature exfoliation of deciduous teeth according to the latest reports, which may help clinicians to form a differential diagnosis.

## 2. Materials and Methods

**Figure 1:**
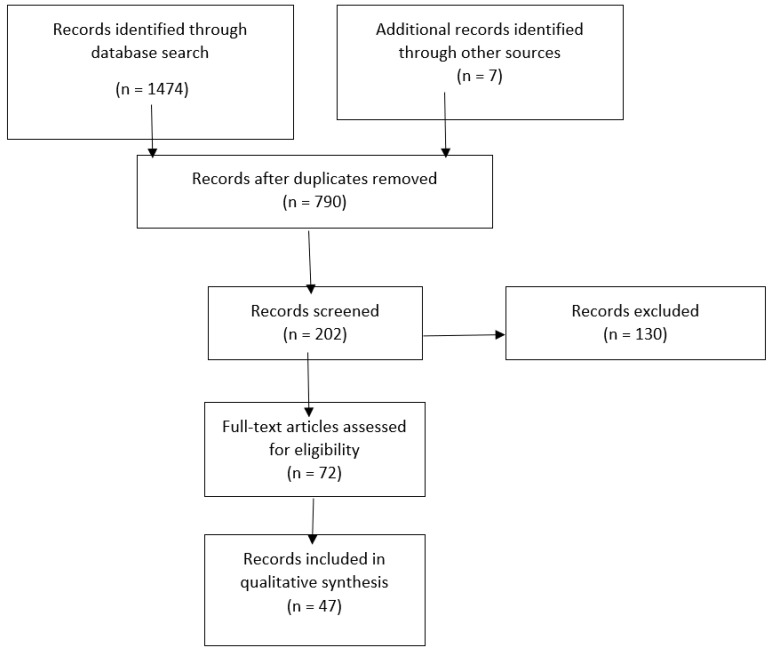
PRISMA flow diagram.

This study is a narrative review of original studies and case reports published in English and Polish between 1957 and 2021 that was conducted by searching the electronic scientific databases PubMed, Google Scholar, Web of Science, and Science Direct to find relevant data, utilizing medical keywords such as tooth loss, systemic disease, deciduous tooth loss, and premature exfoliation of deciduous teeth. The schema of the qualification process was represented by a Preferred Reporting Items for Systematic Reviews and Meta-Analyses (PRISMA)—Figure 1. The search was complemented manually by references from the most recent reviews and case reports. The titles of the papers were verified, and duplicates were excluded. In total, 202 articles were identified; 129 articles were excluded after provisional assessment of the titles and abstracts by two reviewers, and 67 articles were excluded after full-text screening. Finally, 47 articles were found to be relevant to the topic, including 1 review, 18 original papers, and 26 case reports regarding systemic disease resulting in premature tooth loss.

## 3. Results

After compiling information from relevant articles, we categorized systemic diseases that result in premature tooth loss in children into three groups: tooth loss due to periodontal breakdown, leukocyte defects, and defective development of tooth cementum—Table 1.

The first group includes all diseases that cause premature tooth loss due to periodontal breakdown. The function of the junctional epithelium is to protect tooth tissue against constant exposure to oral microbes and their by-products. There are many factors involved in adhesion, such as cell–cell interaction, chemotaxis, proinflammatory cytokines, epithelial growth factor, MMP activation, and antimicrobial peptide production. Extended bacterial virulence and inflammation cause apical migration of the junctional epithelium and activation of collagen destruction. The balance between tissue removal and regeneration is disrupted. Tissue degradation by neutrophils, macrophages, and osteoclasts is stimulated. Finally, inflammatory cells degrade collagen in the connective tissue, periodontal ligament, and alveolar bone. As the destruction proceeds, bone loss results in tooth mobility and exfoliation [2].

The second group includes diseases that cause premature tooth loss due to defective development of tooth cementum. Cementum is thin, mineralized tissue covering the root dentin. There are two types of cementum, non-cellular and cellular. Cellular root cementum covers the apical part of the root and plays a role in post-eruptive tooth movement and adaptation to occlusion, while non-cellular cementum covers the cervical part of the root and is necessary for tooth attachment to the adjacent periodontal ligament (PDL). Mutation in the ALPL or RSK2 gene causes defective development of non-cellular cementum or its absence in patients suffering from hypophosphatasia or Coffin–Lowry syndrome, which leads to PDL detachment, tooth mobility, and tooth loss [3].

The last group includes diseases caused by leukocyte defects. Neutrophils constitute the majority of cells recruited to the periodontium. Several functions exist to maintain neutrophil homeostasis, such as granulopoiesis, release from bone marrow, trafficking and transmigration, and clearance of apoptotic neutrophils. In various disorders, mostly of genetic origin, these processes can be dysregulated. In cyclic neutropenia, a reduced absolute neutrophil count in the circulation causes higher susceptibility to infections. A lack of neutrophils in periodontal tissue signifies no “defensive wall” against subgingival biofilm. This leads to early-onset periodontitis. In LAD-1, due to a deficiency of ß2 integrins, leukocytes cannot adhere to vascular endothelial cells, neutrophils cannot be recruited to tissues, and there is no phagocytosis, which leads to recurrent infections. Patients with this condition develop periodontitis in early life, leading to premature tooth exfoliation. In Papillon–Lefèvre, Chediak–Higashi, and Down syndromes, neutrophil chemotaxis and phagocytosis are impaired. Neutrophils cannot reach the infection site in the periodontium, and patients develop severe bone loss in early life [4].

### 3.1. Diseases Associated with Periodontal Breakdown

(1) Papillon–Lefèvre syndrome (PLS) is a rare autosomal disorder of unclear origin characterized by palmoplantar hyperkeratosis and aggressive periodontitis, leading to premature tooth loss at a very young age [5,6]. Affected children may also suffer from intracranial calcifications, increased susceptibility to bacterial infection, and mental retardation [5]. Dermatological lesions manifest as redness on palms and soles with painful fissures [6]. Tooth eruption begins at a normal age, but soon after, periodontal destruction and rapid bone loss lead to premature exfoliation of primary teeth [5,6,7].

Al Barrak et al. presented a case of Papillon–Lefèvre syndrome among five siblings, all with severe bone destruction and abnormal tooth mobility leading to premature loss of deciduous teeth and hyperkeratotic palms. In this report, consanguineous parents were indicated as a probable reason for the children’s disorder [5].

Another paper, by Gungor et al., reported a similar occurrence of PLS in three sisters, all of whom presented well-demarcated hyperkeratotic plaques on hands and alveolar bone loss with premature exfoliation of both primary and permanent teeth [6].

Unfortunately, there is no definitive treatment to stop the resorption of alveolar bone and premature tooth loss. An interdisciplinary approach that includes the dentist, a dermatologist, and a pediatrician is necessary. Conventional periodontal treatment, hygiene instruction, and systemic antibiotics are recommended.

(2) Congenital adrenal hyperplasia (CAH) is an inherited disorder characterized by insufficient production of cortisol. Patients with CAH demonstrate increased bone resorption and increased risk of developing periodontal disease that may be related to premature tooth loss. Angelopoulou reported a case of a 4.5-year-old boy with worn primary dentition with no signs of caries or enamel hypomineralization who presented abnormal tooth mobility. Before the scheduled treatment, the patient lost the lower left central incisor due to bone loss. Histological examination revealed normal tooth structure. The alkaline phosphatase level was within the normal range. The child needed endocrinological consultation, and application of 25(OH) vitamin D3 supplements helped to stop tooth mobility and further bone destruction [8]. It should be recognized that patients with CAH can prematurely lose their deciduous teeth due to bone resorption. No further articles referring to CAH and premature tooth loss were found in the scientific literature.

Vitamin D plays an important role in calcium homeostasis and is associated with increased bone mineral density and reduced risk of fractures. Its deficiency can manifest in the oral cavity as periodontitis, caries, or even premature tooth loss.

Zhan et al. indicated that higher serum 25OHD concentrations are associated with a lower risk of tooth loss [9]. An American study led by Jimenez et al. suggested that either vitamin D itself or its components may contribute to a reduced risk of tooth loss or periodontitis [10]. Therefore, vitamin D supplementation to prevent its deficiency may play a crucial role in children’s normal growth and development as well as prevent oral diseases and premature tooth loss.

(3) Langerhans cell histiocytosis (LHC), formerly called histiocytosis X, is a rare condition with a greater incidence in children, characterized by expanding proliferation and accumulation of Langerhans dendritic cells in one organ [11,12]. Frequently located in bones and skin, it can also be found in other organs [11], and it is categorized as localized or disseminated. Skin lesions may be the first and sometimes the only symptom of the disease, ranging from scaly erythematous lesions to papular and exfoliative lesions. LHC can also affect lymph nodes, liver, spleen, lungs, and bone marrow. In nearly half of all cases, the oral cavity is affected. The gums are inflamed, painful, and swollen. Severe bone resorption causes the characteristic radiological image of “floating teeth in the air”. Both deciduous and permanent teeth tend to exfoliate prematurely.

Martínez et al. reported the case of an 11-month-old child hospitalized for a suspected peritonsillar abscess. Physical examination showed erythematous skin lesions, cervical lymphadenopathy, and a purplish tumor-like lesion that presented necrotic areas intraorally in the area of tooth 73. Teeth 71 and 72 presented pathological mobility. An incisional biopsy was performed for histopathological examination, which confirmed the diagnosis of LHC. Unfortunately, despite receiving treatment, the patient’s condition became more severe, and he ultimately died [11].

Olczak-Kowalczyk et al. presented the case of a 10-month-old boy with tetralogy of Fallot admitted to the Department of Oral Pathology for intraoral examination and elimination of potential focal infections before heart catheterization. The child suffered from recurrent papular skin eruptions and intertrigo in the umbilical and groin areas. The boy had been seen by a dermatologist, but no proper diagnosis was confirmed. Intraoral examination revealed poor oral hygiene, gingivitis, and numerous teeth with dental caries. Teeth 52, 53, 71, 72, 81, and 82 presented with grade 2 mobility. Radiologic examination confirmed large bone destruction in both the maxilla and mandible. The presence of skin lesions coupled with the intraoral examination indicated a diagnosis of Langerhans histiocytosis. Histopathology confirmed the diagnosis. Mobile infected teeth were removed to avoid the harmful effects of odontogenic infection foci. The patient responded well to chemotherapy treatment and remains under medical surveillance [12].

Devi et al. presented the case of a 4-year-old girl admitted to the Department of Oral Surgery complaining of aching gums while eating. Intraoral examination revealed generalized gingival erythema and pathological tooth mobility; some primary teeth had already been lost. Radiological examination showed severe bone loss and “floating” teeth. Histopathological analysis confirmed the diagnosis of LHC. Apart from this, the patient was suffering from diabetes insipidus, which is the most common endocrine problem accompanying LHC [13].

Henry et al. reported the case of a 15-month-old girl suffering from diarrhea, diminished appetite, and ankle swelling. Intraorally, there was a lesion in the left maxillary posterior area surrounding the crown of an erupting first molar. Medical evaluation and gingival biopsy confirmed LHC, and 12 months later, maxillary disease resulted in pathological bone loss. The patient underwent prednisone and vinblastine treatment and has been asymptomatic since [14].

(4) A very rare syndrome known as mucocutaneous dyskeratosis was reported by Agostini et al. This condition presents with numerous papular skin lesions with keratotic plugs on the limbs and erythematous gingival enlargement. One case report of a 16-month-old child described symptoms such as recurrent episodes of red, suppurating, and itching skin lesions on the lower limbs, abdomen, and back that became brownish with time. New hyperkeratotic lesions developed in lines of trauma, demonstrating the Koebner phenomenon. Enlarged, inflamed gingiva covered the crowns of newly erupted teeth that showed severe mobility. Panoramic radiography revealed large alveolar bone loss. A lack of bone support and periodontal breakdown led to the premature loss of deciduous teeth. Since this condition can resemble Papillon–Lefèvre syndrome, it was excluded by the presence of numerous dyskeratotic cells on gingival biopsy [15]. Form et al. reported a similar case of dyskeratosis in a father and son. It was concluded that gingival inflammation during tooth eruption may be a result of masticatory trauma, resembling the Koebner phenomenon on the skin [16]. Only two articles refer to this condition; therefore, it needs further investigation.
(5) A rare episodic disease known as erythromelalgia is a vascular disorder that causes severe burning pain, redness, and inflammation of distal extremities. Attacks occur periodically and are usually triggered by heat or stress. Prabhu et al. reported the case of a child with erythromelalgia who lost deciduous teeth prematurely. Since the periodontal ligament is more vascular than fibrous in children’s dentition, it may speed up periodontal destruction [17,18].
(6) Hajdu–Cheney syndrome (HCS) is a rare genetic disorder caused by a mutation in the NOTCH2 gene characterized by short stature, severe osteoporosis, acro-osteolysis, dysmorphic facial features (mild hypertelorism with telecanthus, low-set ears, long philtrum, short neck), and dental abnormalities (highly arched palate, impaired tooth eruption, premature tooth loss).

Lee et al. reported the case of a 5-year-old girl with early loss of deciduous central incisors. Hematological and radiological examination showed no abnormalities. She had no previous medical problems except for planovalgus and hyperflexibility of the hands and feet. Molecular genetic examination confirmed the diagnosis of CHS [19].

(7) Short bowel syndrome (SBS) is a rare disorder caused by congenital shortening of the small intestine or acute illness such as necrotizing enterocolitis, necessitating massive intestinal resection. Metabolic consequences, such as vitamin and mineral malabsorption, depend on the remaining amount of absorptive surface. In severe cases, patients can develop osteomalacia, osteoporosis, or spontaneous bone fractures.

Wright et al. reported the case of a well-developed 2-year-old boy who was referred for consultation due to loose teeth. The patient suffered from SBS secondary to necrotizing enterocolitis, chronic hyponatremia, gastrostomy with gastric tube, febrile seizures, anemia, and recurrent Broviac catheter sepsis. Oral examination revealed complete primary dentition free of caries, mild marginal gingivitis, and bleeding on probing. Maxillary central incisors exhibited grade 2 mobility. Radiologically, there was moderate bone loss in the anterior maxillary area. Shortly thereafter, the patient lost his left deciduous incisor while eating.

Presumably, SBS and its associated electrolytic calcium/phosphorus imbalance leads to alveolar bone loss. Lack of mastication (total parenteral nutrition) and poor oral hygiene can also be contributing factors. No more articles were identified regarding the link between SBS and premature tooth loss; therefore, further investigation is needed [20].

(8) Cherubism is a benign autoinflammatory bone disease limited to the mandible and maxilla. As a medical condition, it was first described by Jones in 1933 as a “familiar, multiocular cystic disease of the jaws” [21]. Soon after, it was renamed cherubism, since affected children resemble cherubs, chubby angelic children depicted in Renaissance paintings. It is a rare genetic disorder inherited in an autosomal-dominant manner. Diagnosed children may have an affected parent, but most of them develop the disorder due to a de novo pathogenic variant. This disease usually starts between 2 and 7 years of age with rapid bone degradation restricted to the jaws. It should be noted that mandibular cherubism is more frequently observed than maxillary. Bone loss is replaced by symmetric cystic changes filled with fibrous tissue mass consisting of stromal cells and osteoclast-like cells. As a result, the patient’s face becomes enlarged and disproportionate. In most cases it is considered a benign, self-limited condition and may be clinically unrecognizable; however, in severe cases, bone degradation can result in numerous dental, swallowing, speech, vision, and respiratory complications. The oral manifestation of cherubism is observed as jaw overgrowth with displaced, unerupted, or absent teeth. Premature exfoliation of deciduous teeth is frequently noted. Radiologically, radiolucent multilocular lesions can be seen on both sides of the maxillary and mandibular bones; teeth are displaced and may appear to be floating in cyst-like spaces. Massive enlargement of the jaws can be extremely painful, causing swallowing or respiratory problems, which can lead to surgical intervention. In most cases, patients have an uncomplicated course; the disease progresses until puberty, then starts to decline. By the age of 30, jaw abnormalities are no longer present [21]. Children with cherubism should be supervised and require the interdisciplinary medical cooperation of a dentist, surgeon, orthodontist, ophthalmologist, and speech therapist.

### 3.2. Diseases Associated with Defective Development of Root Cementum

(9) Hypophosphatasia is a rare dominant or recessive disorder caused by a mutation in the tissue non-specific alkaline phosphatase gene. Alkaline phosphatase deficiency disturbs osteogenesis and cementogenesis. Cementum hypoplasia or aplasia and bone degradation result in early tooth loss without clinical signs of root resorption [22]. Symptoms vary from life-threatening to bone pain, leg bowing, skeletal deformities, delayed walking, short stature, recurrent fractures, muscular insufficiency, or premature tooth loss. Biochemical testing reveals decreased levels of serum alkaline phosphatase (APase), and urine analysis shows elevated phosphoethanolamine (PEA) levels [23]. There are six clinical forms of hypophosphatasia: perinatal, prenatal, infantile, childhood, adult, and odontohypophosphatasia (where the only clinical manifestation is premature tooth exfoliation). Fraser reported that 75% of children with skeletal symptoms experience premature exfoliation of deciduous teeth [24]; therefore, it may be the first recognizable “trigger” sign of hypophosphatasia. A characteristic sign of this disorder is a lack of root resorption. Before shedding, teeth exhibit increased pathological mobility [19,20].

Cheung et al. reported two separate cases of 15- and 21-month-old children, both healthy, well-developed, and well-nourished, with premature exfoliation of non-resorbed primary teeth. They both had elevated urine PEA levels and APase levels below the normal range. Both mothers were also biochemically tested, and both had subnormal APase levels and elevated PEA levels in urine. It was concluded that the mothers were heterozygous carriers of this condition [23].

Hollis et al. reported the case of a healthy 20-month-old girl with pathological front tooth mobility (71,81) that increased over time. Oral hygiene was optimal. Radiologic examination revealed severe bone resorption in the area of teeth 71 and 81. The biochemical test detected no abnormalities. Plain radiographs of her wrists and knees did not show any mineralization defects. Microscopic examination of exfoliated teeth revealed no acellular or cellular cementum on the radicular dentin. The results of repeated sampling of serum APase revealed levels at the lower limit of normal range, which confirmed the diagnosis of hypophosphatasia [25].

Illington et al. reported the case of 2-year-old boy with premature exfoliation of deciduous teeth from unknown causes. By the age of 3, there were only seven primary teeth left in his mouth. An X-ray of the tibia revealed bowing on both sides and an area of sclerosis at the upper end of the diaphysis. Similar changes were present in other long bones. Serum APase levels were low [26]. No diagnosis was made at that time. Presumably, this was another case of childhood hypophosphatasia.

Hughes et al. reported the case of a 4-year-old boy referred to the Department of Pediatric Dentistry who experienced premature exfoliation of his primary teeth. Despite numerous medical investigations, no diagnosis was confirmed. The first tooth erupted by the age of 15 months, became mobile, and exfoliated shortly afterwards. All 11 primary anterior teeth exfoliated after eruption, causing difficulties with speech and mastication. Intraoral examination revealed healthy mucosa, caries-free dentition, and good oral hygiene. The roots of exfoliated teeth exhibited a small degree of root resorption. Biochemical tests revealed a low APase level, supported by elevated PEA in the urine and the presence of radiolucency in the metaphyseal area of the bones, confirming the diagnosis of childhood hypophosphatasia [27]. The childhood form of hypophosphatasia usually develops after 6 months of age and is characterized by short stature, waddling gait, and skeletal deformities. Since there is no accepted medical treatment for this condition, removable partial dentures can be used to improve aesthetics, mastication, and speech.

Feeney et al. reported the case of a healthy 2-year-old child with early exfoliation of his primary incisors. Radiological examination revealed bulbous crowns with large pulp chambers and vertical bone loss. Blood tests revealed vitamin D deficiency and low APase levels. Two years later, the child lost all four primary incisors, and the rest of the dentition was mobile. After being referred to the metabolic bone diseases team, the child was diagnosed with odontohypophosphatasia [28].

Thomson et al. reported a case of siblings with premature loss of milk teeth and mobility in the remaining dentition. Biochemical tests revealed decreased APase levels and increased CRP levels. Both were diagnosed with odontophosphatasia [29].

Mori et al. presented nine cases of adult patients (33 to 70 years of age), among whom eight experienced premature loss of deciduous teeth during early childhood. Most of them went without a hypophosphatasia diagnosis for a long time and suffered from multiple recurrent fractures and chronic muscle/joint pain, leading to deterioration in quality of life [30]. This indicates that it is highly important to thoroughly diagnose any kind of suspicious tooth exfoliation. If those patients had received a diagnosis of hypophosphatasia earlier, they would have received the necessary medical treatment to enable an improved quality of life.

(10) Coffin–Lowry syndrome is a rare genetic disorder. Affected children show signs of mental retardation, short stature, thick hands with tapering fingers, and characteristic facial features such as marked hypertelorism, thick lips, large nose with broad base, short upturned nostrils, and protuberant ears [31,32,33]. Hartsfield et al. suggested that sensorineural hearing deficits may also be a manifestation of CLS [31]. Oral manifestations include hypodontia, reduced crown form, narrow palate, midline lingual furrow, delayed eruption, and early tooth loss [31,32].

Igari et al. presented the case of a 5-year-old boy with all anterior teeth (52–82) exfoliating one-by-one beginning at the age of 3. In this case, the teeth showed elongation, root exposure, and rapid root resorption prior to exfoliation. The teeth were free of dental caries. Alkaline phosphatase levels were within normal range [32]. Nordefryd et al. reported the case of a 3-year-old boy with premature loss of primary incisors without preceding root resorption. The teeth were caries-free. Radiographs revealed alveolar bone loss around the remaining incisors. Alkaline phosphatase levels were within normal range. Histological analysis showed hypoplastic root cementum [33]. The two boys had different mechanisms of tooth exfoliation. Despite this, pediatric dentists were the first to diagnose Coffin–Lowry syndrome, since early tooth loss in this age group may be particularly indicative of this disease. Other symptoms become more marked with increasing age.

### 3.3. Diseases Caused by Leukocyte Defects

(11) Chediak–Higashi syndrome is a rare autosomal disorder characterized by large lysosomal granules in granulocytes caused by mutations in the lysosomal trafficking regulator (LYST) gene. In this syndrome, neutrophils and monocytes are defective in chemotaxis, bactericidal, and degranulation capability and are hyperactive in their phagocytic capacity. From infancy, children suffer recurrent infections involving the skin and respiratory system, such as periorbital cellulitis, otitis media, pneumonia, pyoderma, abscesses, sinus infections, and dental caries [34,35]. According to the literature, clinical findings such as hair color (silvery gray) and hematologic abnormalities (e.g., pancytopenia) may be typical in CHS. Periodontal disease may appear as a consequence of systemic alterations and manifests as severe periodontitis with premature loss of primary teeth and generalized or local bone resorption [35].

Rezende et al. presented the case of a 10-year-old boy with intense gingival inflammation, attachment loss, bone resorption, and pathological mobility of all deciduous teeth. The teeth were caries-free and gingival tissues were swollen and bled on probing. Panoramic radiography revealed extensive alveolar bone loss. The patient had decreased pigmentation of his hair and eyes. Blood examination confirmed the diagnosis of Chediak–Higashi syndrome [35].

(12) Marcio da Fonesca et al. reported a case of early tooth loss due to cyclic neutropenia, a transient decrease in neutrophil count. The patient, by the age of 2 years, suffered from severe gingivitis, gingival bleeding, and loose primary teeth. By the age of 5, the only remaining teeth were the canines and the maxillary right second molar. Systemic manifestations of the disease included fever, malaise, and upper respiratory tract infections that led to multiple hospitalizations. Unfortunately, patients with cyclic neutropenia are prone to bacterial infection, leading to fast periodontal destruction, alveolar bone loss, and premature exfoliation of primary and permanent teeth [36].
(13) Down syndrome is a genetic disorder caused by the presence of a third copy of chromosome 21. It is one of the most common causes of mental handicap in children. It is usually diagnosed prenatally or soon after birth based on findings of upslanted palpebral fissures, a flat facial profile, hypotonia, and joint hyperflexibility. Congenital heart disease and abnormalities of the alimentary tract may also be present. Affected children are very susceptible to periodontal disease, often with generalized and rapid periodontal pocket formation and premature tooth loss. These oral problems may come from the effect of mental retardation on these patients’ ability to maintain proper oral hygiene; however, poor neutrophil chemotaxis and phagocytosis also play crucial roles in the periodontium condition [1,34,37].
(14) Acatalasia (acatalasemia or Takahara disease) is an inherited autosomal disorder caused by the absence or very low levels of the enzyme catalase caused by a mutation in the CAT gene. Catalase regulates H_2_O_2_ concentration by metabolizing this molecule into oxygen and water. This reaction protects cells from oxidative assault, for example, by safeguarding pancreatic ß cells against hydrogen peroxide injury [38]. Affected patients suffer from severe periodontitis, necrosis, and ulceration of soft and hard tissue. If their blood comes into contact with hydrogen peroxide, it turns brown, and there is no “bubbling” phenomenon.

Wang et al. reported the case of a well-developed 10-year-old boy with severe gingival pain and swelling. His parents stated that he suffered from recurrent gingival swelling, pain, ulcers, and necrosis, which led to mobility and premature loss of deciduous teeth. Panoramic radiographs revealed alveolar bone loss. Venous blood was collected and genomic DNA was extracted, confirming the diagnosis of acatalasia. It is assumed that in acatalasic patients, bacteria present in tooth crevices produce H_2_O_2_ that cannot be decomposed, leading to the development of gangrenous disease [39].

Delgado et al. reported a case of two brothers who had experienced loose teeth, premature exfoliation of primary teeth, recurrent ulceration, and necrosis of the gingiva. Upon application of hydrogen peroxide, the tissue turned brown-black, and no foam appeared, indicating acatalasia. Blood tests confirmed the diagnosis [40].

(15) Leukocyte adhesion deficiency (LAD) type I is a rare immunodeficiency disorder characterized by defects in integrin receptors of white blood cells. It includes impaired adhesion and chemotaxis, accompanied by increased susceptibility to infections. There are three types of LAD. LAD1 is a disorder that involves deficiencies in three-membrane integrins, which prevents neutrophils from adhering to vessel walls at sites of infection. In spite of leukocytosis, neutrophils cannot migrate into infected tissue. Clinically, it presents as ulceration and tissue necrosis [4,41].

Waldrop et al. reported a case of a family suffering from LAD1. Two children presented with acute gingival inflammation of both primary and permanent dentition, gingival hyperplasia, recession, tooth mobility, and pathologic migration. Primary teeth were lost by the age of 4 [42].

Dababneh et al. reported the case of a 10-year-old girl referred to a periodontology clinic complaining of bleeding gums and loose teeth. The patient had a history of recurrent skin infections, otitis media, oral ulcerations, oral candidiasis, and periorbital cellulitis. Blood tests revealed leukocytosis with mild anemia. The patient was referred to a pediatric immunologist, who diagnosed LAD1. When the girl was 7, she lost her two primary upper incisors because of hypermobility. Oral examination revealed severe gingival inflammation with loose dentition. The patient received initial-phase periodontal therapy and was recalled for monthly prophylaxis to avoid further tooth loss [43].

(16) Wiedemann–Steiner syndrome is a rare autosomal-dominant syndrome that causes developmental delay, dysmorphic face, skeletal features, pre–postnatal growth deficiency, and reduced muscle tone. Dental findings include premature loss of primary teeth, hypodontia, widely spaced dentition, malocclusion, supernumerary teeth, and cleft palate. Hirst et al. reported the case of a 7-year-old girl who was referred to dental clinic for orthodontic consultation. Extraorally, the child showed distinctive facial features such as telecanthus, long and narrow palpebral fissures, smooth long philtrum, and generalized hypertrichosis. She also had developmental delay, and there were concerns that she exhibited autistic spectrum features as well. The interview revealed that all of her primary teeth erupted by the age of 8 months, and most of them exfoliated by 36 months. The child’s oral hygiene was good, and her teeth were caries-free. At the age of 6 years and 9 months, the girl had only permanent teeth. Orthodontic treatment was planned, and the child remains under dental surveillance [44].

Shepard et al. performed a study of 104 individuals with WSS to characterize the syndrome’s clinical and molecular spectrum in a diverse population. They performed bone X-rays, and one in four patients showed advanced bone age [45]. Miyake et al. identified six novel KMT2A mutations in six patients suffering from WSS with skeletal abnormalities, growth impairment, and abnormal dentition (early loss of primary teeth, premature dental eruption). Some of them also showed advanced bone age [46]. There may be a correlation between advanced bone age and premature exfoliation of primary teeth; this topic needs to be further investigated.

## 4. Conclusions

There are many consequences of premature exfoliation of deciduous teeth, including articulatory, orthodontic, and psychosocial disorders. Premature loss of primary dentition can affect children’s phonation, causing speech distortion. The maintenance of deciduous arch integrity has a strong influence on the development of permanent successors. Malocclusions are associated with early tooth loss and often require orthodontic treatment such as space maintainers. Last but not least, psychosocial effects may have a crucial role in children’s development. Quality of life, aesthetics, and the social life of children are negatively impacted by premature tooth loss. These effects can be minimized by using fixed aesthetic space maintainers that replace missing anterior teeth.

Pathological mobility and premature exfoliation of primary teeth should always prompt clinicians to perform a thorough investigation. Proper diagnosis is not easily done and requires multidisciplinary collaboration between doctors and dentists. In such cases, complete medical investigations, such as blood and urine analysis, and sometimes gingival biopsy, should be performed to exclude underlying systemic disorders and select the best possible treatment option. In many cases of premature tooth loss, a group that includes an internist, pediatrician, pediatric dentist, orthodontist, and speech therapist needs to be engaged. Forming a group of people with different approaches and skill levels is very important, so that the case can be viewed from different perspectives. A collaborative practice, shared roles and responsibilities, and, most of all, communication among professionals are necessary to achieve long-term therapeutic success [47].

## Figures and Tables

**Table 1:** Mechanisms of premature deciduous tooth loss due to systemic disease.

Mechanism of Premature Tooth Loss	Disease (Cause of Disease)
Periodontal Ligament Destruction	
Periodontitis combined with chemotaxis deficit	Papillon–Lefèvre syndrome (mutation of cathepsin C gene)
Periodontitis due to vascular alterations	Erythromelalgia (hyperexcitability of C-fibers in dorsal root ganglion, medication-induced)
Periodontitis due to insufficient cortisol production	Congenital adrenal hyperplasia (mutation of 21-hydroxylase gene)
Periodontitis due to accumulation of Langerhans cells	Langerhans cell histiocytosis (mutation of several genes in MAPkinase pathway)
Periodontitis due to metabolic malabsorption	Short bowel syndrome (surgical removal of a portion of small intestine)
Periodontitis due to quick cellular aging	Mucocutaneous dyskeratosis (mutations in TERT, TERC, DKC1, and TINF2 genes)
Periodontitis due to abnormalities in osteoblast and osteoid function	Hajdu–Cheney syndrome (mutation in NOTCH2 gene)
Periodontitis due to osteoclastic degeneration in jaws	Cherubism (mutation in SH3BP2 gene on chromosome 4)
Defective development of non-cellular cementum	Hypophosphatasia (mutation in ALPL gene)
	Coffin–Lowry syndrome (mutation in RSK2 gene)
Leukocyte defects	
Decreased production and release of neutrophils	Cyclic neutropenia (mutation in ELANE gene)
Impaired adherence to vascular endothelium and impaired phagocytosis	Leukocyte adhesion deficiency type I (mutation in ITGB2 gene)
Deficits in chemotaxis and phagocytosis	Down syndrome (trisomy of chromosome 21)
Altered migration, degranulation, and phagocytosis	Chèdiak–Higashi syndrome (mutation in LYST gene)
Catalase deficiency	Acatalasia (mutation in CAT gene)
